# PLSCR3 Deficiency Triggers mtDNA‐Driven cGAS‐STING Activation to Potentiate Antitumor Immunity in Colorectal Cancer

**DOI:** 10.1155/humu/8545428

**Published:** 2026-05-25

**Authors:** Limian Ling, Jingyu Wu, Lei Bao, Zhaohui Liu, Qun Deng, Xiaowen Ji, Shaojun Yu, Feng Yu, Akao Zhu, Qian Xiao, Wenwen Zheng

**Affiliations:** ^1^ Department of Colorectal Surgery and Oncology (Key Laboratory of Cancer Prevention and Intervention, China National Ministry of Education), The Second Affiliated Hospital, Zhejiang University School of Medicine, Hangzhou, Zhejiang, China, zju.edu.cn; ^2^ Zhejiang Cancer Hospital, Hangzhou Institute of Medicine (HIM), Chinese Academy of Sciences, Hangzhou, Zhejiang, China, cas.cn; ^3^ Operating Room, General Hospital of Medical Group in Pingzhuang Mining Area, Chifeng, Inner Mongolia, China; ^4^ Department of Anorectal Surgery, First People′s Hospital of Yuhang District, Hangzhou, Zhejiang, China; ^5^ Department of Surgery, Longquan People′s Hospital, Lishui, Zhejiang, China; ^6^ Cancer Institute (Key Laboratory of Cancer Prevention and Intervention, China National Ministry of Education), The Second Affiliated Hospital, Zhejiang University School of Medicine, Hangzhou, Zhejiang, China, zju.edu.cn

**Keywords:** anti-PD-1, cGAS-STING pathway, colorectal cancer (CRC), immunotherapy resistance, microsatellite-stable (MSS), mitochondrial DNA (mtDNA), mitochondrial integrity, PLSCR3, tumor immunity

## Abstract

**Background:**

Immunotherapy efficacy in colorectal cancer (CRC) is largely restricted to microsatellite instability‐high (MSI‐H) tumors, highlighting an urgent need to overcome resistance in microsatellite‐stable (MSS) CRC. Mitochondrial DNA (mtDNA) leakage activates the cGAS‐STING pathway, a potent inducer of antitumor immunity. However, endogenous regulators constraining mtDNA release in CRC, particularly within the inner mitochondrial membrane (IMM), remain poorly defined.

**Methods:**

In CRC cohorts from TCGA, integrated bioinformatics analysis identified dysregulated mitochondria‐associated genes exhibiting significant differential expression and survival outcomes. Phospholipid Scramblase 3 (PLSCR3) emerged as the prime candidate. Functional validation employed siRNA knockdown and CRISPR/Cas9 knockout in human (HT29) and mouse (CT26) CRC cell lines. Mitochondrial integrity was evaluated via JC‐1 membrane potential assay, oxygen consumption rate (OCR) measurement, and cytosolic mtDNA quantification. cGAS‐STING activation was measured by 2 ^′^3 ^′^‐cGAMP ELISA, cytokine (IFN*β* and CXCL10) secretion, immunoblotting (p‐STING and ISGs), and RT‐qPCR. Immune cell cytotoxicity was assessed using ex vivo NK cell killing assays. In vivo antitumor immunity and response to anti‐PD‐1 therapy were evaluated in syngeneic CT26 graft models in BALB/c mice, with tumor‐infiltrating lymphocytes analyzed by flow cytometry.

**Results:**

PLSCR3 was selected for further study because it fulfilled three criteria simultaneously: differential expression in CRC, significant association with survival in univariate analysis, and established mitochondrial localization. PLSCR3 deficiency disrupted mitochondrial integrity, causing membrane depolarization, impaired respiration, and significant cytosolic mtDNA accumulation. This mtDNA leakage robustly activated the cGAS‐STING pathway, evidenced by increased 2 ^′^3 ^′^‐cGAMP, IFN*β*/CXCL10 secretion, STING phosphorylation (Ser366), and ISG upregulation. Functionally, PLSCR3 knockdown enhanced NK cell–mediated killing of CRC cells in vitro. Critically, PLSCR3 knockout in CT26 tumors significantly potentiated the efficacy of anti‐PD‐1 therapy in vivo, overcoming inherent resistance in this MSS‐CRC model. This was associated with increased intratumoral infiltration of CD8+ and CD4+ T cells, elevated Granzyme B levels, and enhanced activation markers on CD8+ T cells within the tumor microenvironment.

**Conclusion:**

Our study identifies PLSCR3 as a previously unrecognized negative regulator of mtDNA‐associated cGAS‐STING activation in CRC. By maintaining mitochondrial homeostasis and limiting mtDNA leakage, PLSCR3 constrains innate immune signaling and reduces sensitivity to anti‐PD‐1 therapy in the CT26 model. These findings establish PLSCR3 as a promising therapeutic target to enhance immunotherapy responses in CRC.

## 1. Introduction

Colorectal cancer (CRC) persists as a leading cause of global cancer morbidity and mortality, posing substantial clinical challenges despite advances in surgery, chemotherapy, and targeted therapies [1, 2]. In recent years, immunotherapy, particularly immune checkpoint blockade (ICB) targeting Programmed Cell Death Protein 1 (PD‐1), has emerged as a transformative modality in CRC [3]. However, only a subset of patients, mostly those with microsatellite instability‐high (MSI‐H) tumors, exhibit durable responses to immunotherapy, highlighting the urgent need to elucidate novel regulatory mechanisms of tumor immunity and identify new therapeutic targets [4, 5].

Mitochondria are central not only to cellular energy metabolism but also to immune regulation and tumor biology [6–8]. Mitochondrial dysfunction and membrane permeabilization provoke leakage of mitochondrial DNA (mtDNA)—a potent damage‐associated molecular pattern (DAMP)—into the cytosol [9–11]. Cytosolic mtDNA is sensed by cyclic GMP‐AMP synthase (cGAS), which catalyzes stimulator of interferon genes (STING)–dependent Type I interferon (IFN‐I) production [12]. Activation of the cGAS‐STING pathway in tumor cells and the tumor microenvironment (TME) has been shown to enhance antitumor immunity and improve responses to ICB [13–15].

Phospholipid Scramblase 3 (PLSCR3), an inner mitochondrial membrane (IMM)–resident lipid transporter, maintains mitochondrial membrane asymmetry and function [16, 17]. Although studies in noncancer contexts implicate PLSCR3 in mitochondrial quality control and mitophagy [18, 19], its involvement in tumor biology and immune regulation has not been thoroughly investigated. Crucially, while outer mitochondrial membrane (OMM) regulators (e.g., PINK1/Parkin) modulate mitochondrial quality control [20], whether IMM gatekeepers such as PLSCR3 constrain antitumor immune responses remains an unresolved enigma.

In this study, we first performed integrative bioinformatic analyses using The Cancer Genome Atlas (TCGA) datasets to identify mitochondria‐related genes differentially expressed in CRC and associated with patient prognosis. Among candidate genes, PLSCR3 emerged as a top candidate, displaying high tumor expression correlated with poor survival. Mechanistically, PLSCR3 deficiency triggers mitochondrial dysfunction, causing cytosolic mtDNA accumulation and cGAS‐STING pathway activation. Functional studies demonstrate that PLSCR3 loss markedly enhances IFN‐I responses and secretion of chemokines that potentiate natural killer (NK) cell and cytotoxic T cell–mediated antitumor immunity. Importantly, PLSCR3 deficiency sensitizes tumors to anti‐PD‐1 immunotherapy in vivo. Critically, PLSCR3 deficiency overcomes PD‐1 blockade resistance in MSS‐CRC in vivo.

In this study, we performed an integrative analysis of CRC transcriptomic datasets to identify mitochondria‐associated genes linked to differential expression and survival. PLSCR3 was prioritized because it was upregulated in CRC, associated with poorer survival in univariate analysis, and localized to mitochondria. Mechanistically, we show that PLSCR3 deficiency impairs mitochondrial homeostasis, promotes mtDNA release into the cytosol, and activates cGAS‐STING signaling. Functionally, PLSCR3 loss enhances immune cell–mediated tumor killing and improves the response to anti‐PD‐1 therapy in a syngeneic CT26 model. These findings support PLSCR3 as a candidate regulator connecting mitochondrial homeostasis to tumor‐intrinsic innate immune signaling in CRC.

## 2. Method

### 2.1. Data Acquisition and Processing

Transcriptomic data and clinical information for COAD‐READ were obtained from TCGA. Raw data were downloaded and processed/filtered using R software (Version 4.0.5). The mitochondria‐related gene set was curated from the MitoCarta 3.0 database (https://www.broadinstitute.org/mitocarta/mitocarta30-inventory-mammalian-mitochondrial-proteins-and-pathways) and GSEA/MSigDB (http://www.gsea-msigdb.org/gsea/index.jsp); the analytical strategy was consistent with previous CRC mitochondrial–gene studies [21]. CRC immunohistochemistry (IHC) images, subcellular localization data, and CRC cell line transcriptomes were sourced from the Human Protein Atlas (HPA) database (https://www.proteinatlas.org/). Healthy human tissue data were acquired from the Genotype‐Tissue Expression (GTEx) Project (https://gtexportal.org/). Prognostic analysis was performed using the KM Plotter online platform (https://kmplot.com/analysis/).

### 2.2. Functional Analysis

Following acquisition of CRC transcriptomic data, differentially expressed genes (DEGs) were identified using the limma package in R (FDR‐adjusted *p* < 0.05, |log₂FC| > 0.585). Subsequent functional annotation of DEGs was conducted through GO (Gene Ontology) enrichment and KEGG (Kyoto Encyclopedia of Genes and Genomes) pathway analyses via the clusterProfiler R package.

### 2.3. Prognostic Analysis

The mitochondria‐related gene set (sourced as above) was intersected with CRC DEGs using R‐based analysis. Genes overlapping between these two sets were subjected to univariate Cox proportional hazards regression utilizing the survival R package. Hazard ratios (HRs) and 95% confidence intervals (CIs) were calculated to evaluate associations with overall survival (OS) in CRC cohorts.

### 2.4. Transfection and Stable Cell Line Construction

Lipofectamine 3000 was used for the transient transfection of plasmids or siRNA duplexes according to the manufacturer′s instructions. For RNA interference, the transfection was repeated twice with an interval of 24 h to achieve the maximal RNAi efficacy. siRNA specific for PLSCR3 (5 ^′^‐AUGGAAGUACAGGCUCCACC‐3 ^′^) was obtained from GentleGen. The stable cell lines were generated by transient transfection of the specific plasmid and selected with G418 or puromycin. PLSCR3 knockdown in HT29 and DLD1 cells was achieved using siRNA.

### 2.5. Lentivirus‐Mediated Gene Knockout in CT26.WT Cells

CRISPR/Cas9‐mediated PLSCR3 knockout was performed in CT26 cells. Briefly, a lentiviral vector expressing gRNA was transfected with package vectors into HEK293T package cells. The virus supernatants were harvested and filtered with a 0.22‐mm filter at 48 and 72 h after transfection. The target cells were infected twice and sorted via flow cytometry–mediated cell sorting. For some experiments, a single cell was plated into a 96‐well plate by flow cytometry for single clone isolation. The isolated single clones were verified by Western blotting.

### 2.6. Cell Lines

The human colon carcinoma cell line HT29 (Cat. No. ATCC‐HTB‐38) and the mouse colon carcinoma cell line CT26.WT (Cat. No. CRL‐2638, ATCC, Virginia, United States) were maintained in RPMI 1640 medium (Cat. No. BC‐M‐017, Bio‐Channel, Jiangsu, China) supplemented with 10% fetal bovine serum. The cell lines were cultured in a humidified atmosphere in 5% CO_2_ at 37°C. Phosphate‐buffered saline (PBS) used in cell culturing was purchased from Bio‐Channel (Cat. No. BC‐BPBS‐01, Bio‐Channel, Jiangsu, China).

### 2.7. Antibodies and Reagents

Anti‐PLSCR3 (mouse/IgG, Cat. No. sc‐100808) was purchased from Santa Cruz Biotechnology (Texas, United States). Anti‐STING (rabbit/IgG, Cat. No. 19851‐1‐AP), anti‐ISG60 (rabbit/IgG, Cat. No. 15201‐1‐AP), anti‐GAPDH (rabbit/IgG, Cat. No. 10494‐1‐AP), and anti‐IFIH1 (rabbit/IgG, Cat. No. 21775‐1‐AP) antibodies were purchased from Proteintech (Illinois, United States). Anti‐Phospho‐STING (rabbit/IgG, Cat. No. 50907s) antibodies were acquired from Cell Signaling Technology (Massachusetts, United States). STING agonist (Cat. No. HY‐103665, CAS No.: 2138299‐29‐1) and DDC (Cat. No. HY‐17392, CAS No.: 7481‐89‐2) were purchased from MedChemExpress (Shanghai, China).

### 2.8. Immunoblot Analysis

Proteins were extracted using RIPA lysis buffer, quantified via BCA assay, and denatured in Laemmli buffer at 95°C for 5 min. Samples (30 *μ*g/lane) were separated by SDS‐PAGE (12% gels) at 120 V and then transferred to PVDF membranes via wet transfer (100 mA, 2 h). Membranes were blocked in 5% nonfat milk/TBST for 1 h at RT, incubated overnight at 4°C with primary antibodies, washed three times with TBST, and probed with horseradish peroxidase (HRP)–conjugated secondary antibodies (1:5000 dilution, 1 h at RT). After final washes, signals were detected using chemiluminescent substrate (ECL) and imaged via digital systems (Azure), with GAPDH as loading controls.

### 2.9. JC‐1 Mitochondrial Membrane Potential Assay

Cells were incubated with JC‐1 dye (MedChemExpress, Cat. No. HY‐K0601) at 37°C in the dark, enabling accumulation in mitochondria; high membrane potential promoted J‐aggregate formation (red fluorescence), while depolarized mitochondria retained JC‐1 monomers (green fluorescence). After staining, cells were washed and analyzed via fluorescence microscopy, with the red/green fluorescence ratio quantified to evaluate mitochondrial membrane potential changes.

### 2.10. JC‐1 Fluorescence Quantification

Mitochondrial membrane potential (*Δ* *Ψ* *m*) was assessed by JC‐1 staining, and fluorescence images were quantified using ImageJ software (NIH). RGB images were split into red and green channels to separate JC‐1 aggregates (red fluorescence, indicating high *Δ* *Ψ* *m*) from JC‐1 monomers (green fluorescence, indicating low *Δ* *Ψ* *m*). Background fluorescence was subtracted using a rolling‐ball radius of 50 pixels. Regions of interest (ROIs) corresponding to cell areas were defined by automated thresholding, and the mean fluorescence intensity (MFI) of the red and green channels was measured within identical ROIs. Mitochondrial membrane potential was expressed as the red‐to‐green fluorescence intensity ratio. All imaging parameters, including laser intensity, exposure time, gain, and objective settings, were kept constant across groups to ensure comparability.

### 2.11. Oxygen Consumption Rate (OCR) Detection

Cells were seeded at 2 × 10^4^ cells per well in Seahorse XF cell culture microplates and allowed to adhere overnight. OCR was measured using a Seahorse XFe96 Extracellular Flux Analyzer (Agilent Technologies, Santa Clara, California, United States). Before the assay, cells were equilibrated in CO_2_‐free Seahorse XF assay medium and sequentially treated with mitochondrial modulators: Oligomycin (1–2 *μ*M) was added first to inhibit ATP synthase, followed by a 10–15‐min measurement of ATP‐linked respiration; FCCP (0.5 *μ*M) was then injected to uncouple mitochondria, with maximal respiration recorded after 2–3 min of equilibration; finally, rotenone (0.5 *μ*M) and Antimycin A (0.5 *μ*M) were coadministered to block Complex I/III, allowing nonmitochondrial oxygen consumption measurement. Oxygen levels were tracked in real time via optical sensors, and OCR values were derived from depletion rates and normalized to cell count or protein content for functional analysis.

### 2.12. Mitochondrial Extraction and Measurement of the D‐Loop mtDNA Ratio

Cells were harvested via centrifugation at 500 × *g* for 5 min at 4°C. The resulting cell pellets were rinsed once with ice‐cold PBS and then resuspended in cold hypotonic buffer supplemented with protease inhibitors (complete protease inhibitor cocktail tablets, Roche). Subsequently, the cells were subjected to lysis using a Dounce homogenizer. The lysates were centrifuged at 500 × *g* for 10 min at 4°C to pellet nuclei (which were discarded), and the supernatants were transferred into fresh tubes. These supernatants were then centrifuged at 5000 × *g* for 10 min at 4°C, yielding two fractions: the mitochondrial fraction (P5K) and the nonmitochondrial cytosolic fraction (S5K). mtDNA was purified from these distinct fractions using a DNA extraction kit (Cat. No. D3396‐01; OMEGA, Norcross, Georgia). Following purification, quantitative real‐time PCR (RT‐qPCR) was conducted using primers specific for the D‐loop region to analyze mtDNA levels. The ratio of mtDNA levels in the S5K fraction to those in the P5K fraction was used to represent the relative release of mtDNA from mitochondria into the cytosol. (D‐loop‐MT325F: 5 ^′^‐CACAGCACTTAAACACATCTCTGC‐3 ^′^; D‐loop‐MT474R: 5 ^′^‐AGTATGGGAGTGRGAGGGRAAAA‐3 ^′^). Because the cytosolic fraction was operationally defined by differential centrifugation, residual mitochondrial contamination cannot be fully excluded and should be considered when interpreting these data.

### 2.13. Quantification of 2^′^3^′^‐cGAMP (Cyclic Guanosine Monophosphate–Adenosine Monophosphate)

To determine the intracellular 2^′^3^′^‐cGAMP level in PLSCR3‐knockdown (KD_PLSCR3) and normal control (NC) HT29 cells, a competitive ELISA was performed using the 2^′^3^′^‐Cyclic GAMP Competitive ELISA Kit (Cat. No. 501700, Cayman Chemical). Briefly, cells were harvested, washed twice with precooled PBS, and lysed in M‐PER lysis buffer (Cat. No. 78501, Thermo Fisher Scientific) on ice for 15 min (with vortex mixing three times); the lysate was then centrifuged at 12,000 × *g* for 15 min at 4°C to collect the supernatant, and protein concentration was normalized using the BCA assay. For ELISA, 100 *μ*L of serially diluted cGAMP standards (0–1600 pg/mL) or samples (in triplicate) were added to antibody‐precoated 96‐well plates, followed by 100 *μ*L of cGAMP‐HRP Tracer; the plate was sealed and incubated at room temperature with shaking (500 rpm) for 1 h. After five washes with 1× wash buffer, 100 *μ*L of TMB substrate was added for 15–20 min of light‐protected incubation, and the reaction was terminated with 50 *μ*L of 2 N H_2_SO_4_. Absorbance at 450 nm was measured using a microplate reader, and the cGAMP concentration was calculated via four‐parameter logistic regression of the standard curve (requiring *R* ^2^ > 0.99). Quality control was ensured by maintaining a coefficient of variation (CV) < 10*%* for standards and < 15% for sample triplicates, with all operations performed on ice or at 4°C to minimize cGAMP degradation.

### 2.14. ELISA

HT29 cells (1 × 10^6^) with or without PLSCR3 knockdown were seeded in six‐well plates in complete growth medium. The secreted chemokines were measured by the Human IFN*β* ELISA Kit (PI572, Beyotime, China) and the Human CXCL10 ELISA Kit (PC208, Beyotime, China), according to the manufacturer′s protocols. Isolated graft tumors were prepared and minced with blades, and then, the tumor tissues were cultured in PBS for 4 h at 37°C. The secreted amount of Granzyme B in the culture was measured by the Mouse Granzyme B ELISA Kit (BMS6029), according to the manufacturer′s protocols.

### 2.15. RT‐qPCR

Total RNA was extracted using TRIzol reagent, quantified via spectrophotometry (A260/A280 ratio ≥ 1.8), and reverse‐transcribed into cDNA using a reverse transcriptase with oligo(dT) or random hexamer primers. qPCR reactions were prepared with SYBR Green Pro Tag HS Premix (AG11701, Accurate Biology, China), gene‐specific primers (100 nM), and cDNA template (50 ng) and then amplified in a real‐time PCR system under standardized cycling conditions (95°C for 10 min, followed by 40 cycles of 95°C for 15 s and 60°C for 1 min). Melting curve analysis confirmed primer specificity, and relative gene expression was quantified via the *Δ* *Δ*Ct method, normalized to endogenous controls (GAPDH), with no‐template and no‐RT controls included to exclude contamination.

### 2.16. Confocal Microscopy

Transfected HT29 cells were subcultured and grown on coverslips. To detect STING status and cellular location, cells were fixed with 4% paraformaldehyde, permeated with Triton‐X100, and incubated in 5% FBS and anti‐STING, followed by incubation with FITC‐conjugated goat antirabbit IgG (H + L) reactivation at room temperature. Cell images were captured by a ZEISS laser scanning confocal microscope (Zeiss LSM 900, Oberkochen, Germany). The ZEISS ZEN Microscope software was used for operation and acquisition. Percentages of foci formation cells were statistically analyzed.

For quantification of STING activation, cells displaying punctate STING foci were counted manually in at least five randomly selected fields per sample under identical imaging settings. The percentage of STING‐positive cells was calculated as the number of cells with STING foci divided by the total number of cells per field. At least 100 cells were analyzed per condition in each experiment. Quantification was performed in three independent biological replicates.

### 2.17. Ex Vivo NK Cell Cytotoxicity

NK cells were isolated from umbilical cord blood (provided by Zhejiang Province Umbilical Cord Blood Hematopoietic Stem Cell Bank). Firstly, umbilical cord blood was diluted and subjected to density gradient centrifugation over a Ficoll layer to isolate peripheral blood mononuclear cells (PBMCs). Subsequently, magnetic‐activated cell sorting (MACS) was employed using a specific NK Cell Isolation Kit (Miltenyi, Cat. No. 130‐092‐657) to selectively enrich for NK cells based on CD56 expression. The enriched NK cell population was further purified through fluorescence‐activated cell sorting (FACS) with targeted antibodies. Finally, the purity and functionality of the isolated NK cells were validated using flow cytometry and functional assays to ensure their integrity and biological relevance.

For cytotoxicity assays, CRC cells (HT29 or DLD1) were seeded in 24‐well plates at a density of 5 × 10^4^ cells per well and allowed to adhere overnight. Purified NK cells were added at an effector‐to‐target (E:T) ratio of 5:1 unless otherwise indicated. Coculture was performed for 24 h at 37°C. After coculture, tumor cell viability was assessed by phase‐contrast microscopy and quantified by counting the number of remaining adherent tumor cells in randomly selected fields (*n* = 5 fields per well). In parallel, cell viability was further confirmed using the trypan blue exclusion assay. All experiments were performed with at least three independent biological replicates.

### 2.18. Preparation of Tumor‐Infiltrating T Cells

Tumors were minced with scissors and then digested with the digestion buffer (RPMI 1640 medium, 5% FBS, 1% penicillin–streptomycin, 25 mM HEPES, and 300 U collagenase, Sigma C0130) on a shaker at 37°C for 2 h. Single cells were prepared through a 70‐*μ*m cell strainer. Erythrocytes were removed by incubation in red blood cell lysis buffer (R7757, Sigma) at room temperature for 5 min. The cells were prepared in PBS (with a concentration of ~2 × 10^7^) for studies.

### 2.19. Flow Cytometry

The single‐cell suspension was fixed with 4% paraformaldehyde solution (E672002, Sangon Biotech, China). CD45^+^ T cells were selected from a single‐cell suspension (about 2 × 10^7^ cells digested from tumor tissue) by flow cytometry with the following antibodies (antimouse CD45; BD Pharmingen, 561875). And then the cells were stained with antimouse CD3e PE (145‐2C11, BD Pharmingen, 553063), antimouse CD4 FITC (RM4‐5, BD Pharmingen, 553046), antimouse CD4 PE/Cy7 (GK1.5, BioLegend, 100421), antimouse CD8 FITC (53‐6.7, BD Pharmingen, 553031), and antimouse CD8a APC/Cy7 (53‐6.7, BioLegend, 100713).

Flow cytometry analysis was performed using a standard gating strategy. Briefly, single cells were first identified based on forward and side scatter profiles (FSC‐A vs. FSC‐H) to exclude doublets. Dead cells were excluded based on viability dye staining when applicable. CD45+ leukocytes were then gated, followed by selection of CD3+ T cells. Within the CD3+ population, CD4+ and CD8+ T cell subsets were further identified. Data were analyzed using FlowJo software.

### 2.20. Syngeneic Colorectal Carcinoma Graft Model

PLSCR3 knockdown or Scramble CT26.WT cells (1.5 × 10^5^)were mixed with BD Matrigel (Matrix Growth Factor Reduced) (BD, 354230) in 100 *μ*L PBS and then subcutaneously injected into the right flanks of BALB/c mice of 8–10 weeks old (from the Jackson Laboratory, 000664). Tumor growth was measured with calipers, and size was expressed in cubic centimeters every 3 days. For antibody treatment, control IgG (10 mg/kg) or anti‐PD‐1 (RMP1‐14, BioXCell, 10 mg/kg) was injected intraperitoneally (i.p.) on Days 6, 9, 12, 15, and 18 after tumor cell inoculation. For the tumor growth curve, grafts were measured with calipers and established (0.5 × length × width^2^) every 5 days. All animal experiments were performed in accordance with the Guide for the Care and Use of Laboratory Animals of the National Institutes of Health, and the protocol was reviewed and approved by the Second Affiliated Hospital of Zhejiang University.

### 2.21. Statistical Analysis

Statistical analyses were performed using GraphPad Prism 11. Data are presented as mean ± SD unless otherwise indicated. Comparisons between two independent groups were performed using an unpaired two‐tailed Student′s *t*‐test. For comparisons involving more than two groups, one‐way ANOVA followed by Tukey′s multiple‐comparison post hoc test was used unless otherwise indicated. Tumor growth curves were analyzed using two‐way repeated‐measures ANOVA with appropriate post hoc multiple‐comparison tests. Differences were considered statistically significant at  ^∗^ *p* < 0.05 and highly significant at  ^∗∗^ *p* < 0.01,  ^∗∗∗^ *p* < 0.001, and  ^∗∗∗∗^ *p* < 0.0001.

## 3. Results

### 3.1. Identification and Analysis of Mitochondria‐Related Prognostic Gene PLSCR3 in CRC

**Figure 1:**
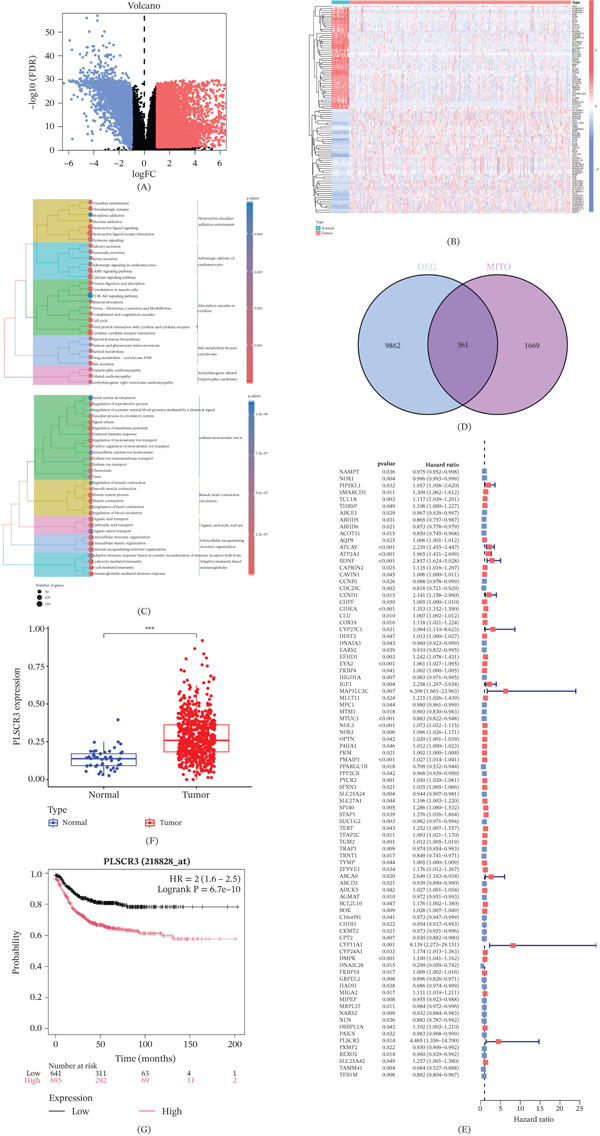
Identification and analysis of mitochondria‐related prognostic gene PLSCR3 in CRC. (A) Volcano plot of DEGs in the TCGA CRC cohort (|log₂FC| > 0.585, adj.*p* < 0.05): upregulated (red), downregulated (blue), and nonsignificant (black). (B) Heatmap of Top 50 significant DEGs: rows = genes, columns = samples (N: normal; T: tumor); color gradient shows expression (red: high; blue: low). (C) Dendrogram of functional enrichment: Branch colors denote functional clusters (yellow box: significant enrichment in metabolic pathways). (D) Venn diagram: DEGs (*n* = 9862, blue) and mitochondrial genes (*n* = 1469, purple); intersection = 363 genes (lavender). (E) Forest plot of prognostic genes: univariate Cox analysis of 89 genes (HR ≠ 1, *p* < 0.05); PLSCR3 highlighted (HR = 2.18, *p* < 0.001, red box). (F) PLSCR3 expression in the TCGA cohort: tumor (red) vs. normal tissues (blue). (G) KM plotter analysis of colon cancer cohort: High PLSCR3 expression (red) correlates with reduced recurrence‐free survival (RFS) (HR = 2.16, *p* = 6.7e − 10).

Transcriptomic analysis of the TCGA CRC cohort identified 10,224 DEGs (log₂FC > 0.585, adj.*p* < 0.05; Figure 1A). To visualize core drivers, the 50 most significantly altered genes were displayed in a clustered heatmap (Figure 1B). Functional enrichment (GO/KEGG) revealed that CRC pathogenesis was significantly associated with metabolic pathways (Figure 1C, yellow‐highlighted module). To investigate mitochondrial involvement, DEGs (blue set, *n* = 9862) were intersected with the MitoCarta 3.0 mitochondrial gene set (purple set, *n* = 1469), yielding 363 mitochondria‐related DEGs (Figure 1D). Univariate Cox analysis identified 89 genes significantly correlated with CRC prognosis. PLSCR3 was prioritized as a key prognostically associated candidate gene due to its high HR (HR = 2.18, *p* < 0.001; Figure 1E). Validation confirmed significantly elevated PLSCR3 expression in CRC tumors versus normal tissues (Figure 1F). High PLSCR3 expression predicted reduced recurrence‐free survival (RFS) in an independent colon adenocarcinoma cohort (KM plotter: HR = 2.16, *p* = 6.7e − 10; Figure 1G).

Analysis of the GTEx database demonstrated ubiquitous expression of PLSCR3 across multiple healthy human organs (Figure S1A). Pan‐cancer profiling further revealed that PLSCR3 was significantly upregulated in > 85% of tumor types (Figure S1B). Both RNA sequencing (nTPM) and protein detection data confirmed consistently high PLSCR3 expression in 63 CRC cell lines (Figure S1C). PLSCR3 expression was markedly elevated in CRC tumors compared to normal tissues (Figure S1D). Crucially, subcellular localization assays unambiguously positioned PLSCR3 within mitochondria (Figure S1E), suggesting its potential role in regulating mitochondrial functions.

PLSCR3 was selected for further study because it fulfilled three criteria simultaneously: differential expression in CRC, significant association with survival in univariate analysis, and established mitochondrial localization.

To further characterize the genomic context of PLSCR3 in CRC, we performed an exploratory analysis of public cancer genomics data. PLSCR3 genomic alterations were relatively infrequent and consisted mainly of missense mutations and deep deletions, indicating that direct genomic disruption of PLSCR3 is not a common event in CRC (Figure S2). Survival analyses based on alteration status suggested a possible association with clinical outcome, although the low frequency of altered cases limited statistical power. These findings suggest that the elevated expression and functional effects of PLSCR3 observed in this study are unlikely to be explained primarily by recurrent genomic alteration and may instead be more closely related to expression level and mitochondrial function.

### 3.2. PLSCR3 Deficiency Disrupts Mitochondrial Homeostasis in CRC Cells

**Figure 2:**
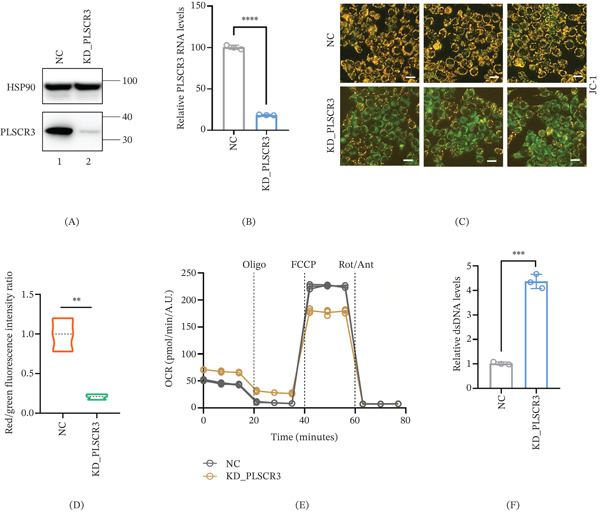
PLSCR3 deficiency disrupts mitochondrial homeostasis in CRC cells. (A) Western blotting verified the reduction of PLSCR3 protein levels in HT29 cells. (B) RT‐qPCR analysis verified reduction of PLSCR3 mRNA levels normalized to GAPDH compared with negative control (NC) cells (*p* < 0.0001, *n* = 3). (C) Representative JC‐1 staining (scale bar: 20 *μ*m) images showing red fluorescence (polarized mitochondria) and green fluorescence (depolarized mitochondria). (D) Quantification of the JC‐1 red/green fluorescence intensity ratio. Fluorescence intensity was quantified using ImageJ software as described in the Method section. (E) Real‐time oxygen consumption rate (OCR) profiles under sequential treatment with oligomycin, FCCP, and rotenone/Antimycin A. (F) Quantification of cytosolic mtDNA by qPCR using the D‐loop ratio in PLSCR3‐knockdown and control cells. Data are presented as mean ± SD from three independent experiments. Statistical significance was determined using an unpaired two‐tailed Student′s *t*‐test for two‐group comparisons.

In this study, “mitochondrial integrity” refers to the preservation of mitochondrial membrane potential, respiratory function, and compartmentalized retention of mtDNA, as assessed by JC‐1 staining, OCR measurement, and cytosolic mtDNA quantification, respectively. The aforementioned results indicate that PLSCR3 expression is upregulated in CRC tissues compared to normal tissues. Therefore, we next sought to investigate the functional role of aberrantly accumulated PLSCR3 in CRC. To investigate the function of PLSCR3 in the mitochondria of tumor cells, we knocked down the expression of PLSCR3 in cells and detected the status of mitochondria. PLSCR3 knockdown in HT29 and DLD1 cells significantly compromised mitochondrial function as evidenced by multiple experimental approaches (Figure 2 and Figure S3). Quantitative Western blotting and RT‐PCR analysis verified over 80% reduction in PLSCR3 RNA levels compared to negative control (NC) (*p* < 0.001, *n* = 3) (Figure 2A,B and Figure S3A). Fluorescence microscopy showed an increase in green JC‐1 monomer fluorescence and a reduction in red aggregate fluorescence in PLSCR3‐knockdown cells compared with control cells (Figure 2C and Figure S3B). Quantitative analysis of the red/green fluorescence intensity ratio further confirmed a significant reduction in mitochondrial membrane potential after PLSCR3 knockdown (Figure 2D and Figure S3C). Mitochondrial respiration analysis demonstrated impaired oxygen consumption capacity under FCCP challenge (*p* < 0.001) (Figure 2E). Concurrently, PLSCR3‐knockdown cells exhibited accumulation of double‐stranded mitochondrial DNA (dsDNA) compared to NC (*p* < 0.001) (Figure 2F and Figure S3D). Together, these findings indicate that PLSCR3 deficiency disrupts mitochondrial homeostasis and promotes mtDNA release in CRC cells.

### 3.3. PLSCR3 Deficiency Activates mtDNA‐Associated cGAS‐STING Signaling

Although some evidence suggests that the cGAS‐STING pathway is difficult to activate due to gene methylation‐mediated expression inhibition in some CRC cells [22], other evidence indicates that in CRC cells with positive cGAS‐STING expression, STING can be activated and exert functions in activating antitumor immunity [23]. Recent studies have shown that mtDNA leakage into the cytoplasm can activate the endogenous cGAS in cells. The activated cGAS produces 2^′^3^′^‐cGAMP, which binds to STING to activate the downstream IFN‐I response. The aforementioned experimental results indicate that knockdown of PLSCR3 disrupts mitochondrial integrity and induces mtDNA leakage in CRC cells. Therefore, the subsequent question we addressed was whether PLSCR3 deficiency activates the endogenous cGAS‐STING innate immune pathway in CRC cells via mtDNA release.

**Figure 3:**
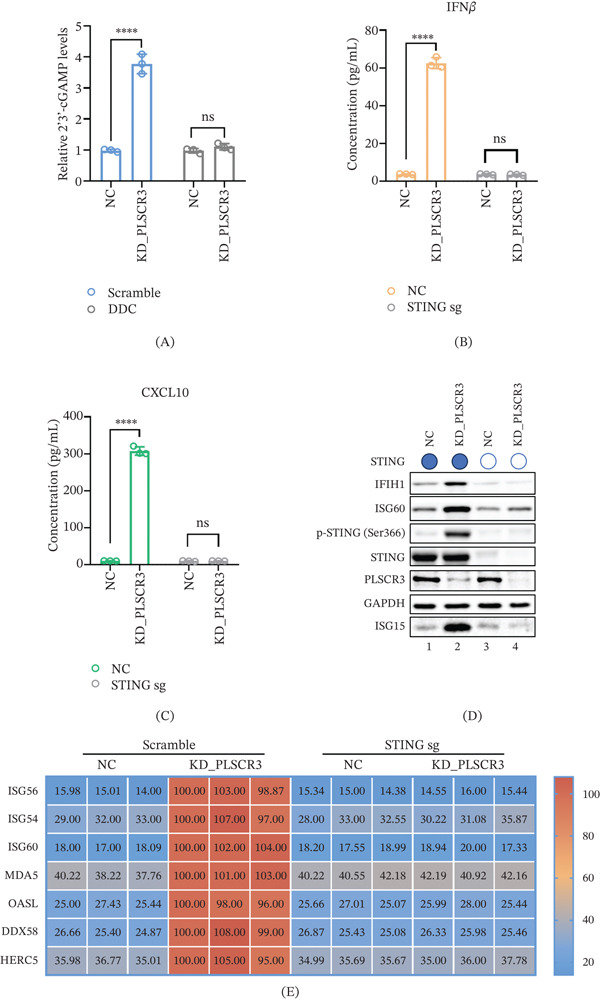
PLSCR3 deficiency activates mtDNA‐associated cGAS‐STING signaling. (A) 2 ^′^‐3 ^′^‐cGAMP quantification by ELISA in PLSCR3 knockdown (KD_PLSCR3) HT29 cells and normal controls (NCs) treated with Scramble or dideoxycytidine (DDC) (*p* < 0.0001; ns = not significant; *n* = 3). (B, C) KD_PLSCR3 cells exhibited an elevation in IFN*β* and CXCL10 production measured by ELISA compared to NC (*p* < 0.0001, *n* = 3). CRISPR‐mediated STING knockout (STING sg) completely abrogated these effects. (D) Western blot analysis showed upregulation of phosphorylated STING and innate immune effectors (IFIH1, ISG60, and ISG15 protein) in STING‐competent KD_PLSCR3 cells relative to GAPDH loading controls. (E) Normalized mRNA level quantification by qPCR demonstrated significant induction of interferon‐stimulated genes (ISGs) in STING‐competent KD_PLSCR3 cells compared with the other groups. Data in panels A–C and E are presented as mean ± SD. Statistical significance was determined using one‐way ANOVA followed by Tukey′s multiple‐comparisons post hoc test for multiple‐group comparisons.

As shown in Figure 3, scramble treatment induced an increase in 2^′^3^′^‐cGAMP levels in PLSCR3‐knockdown cells compared to NCs (*p* < 0.0001; *n* = 3 biological replicates), whereas dideoxycytidine (DDC) treatment—which caused substantial mtDNA depletion—abolished this increase, showing no significant difference from NC (ns; *p* > 0.05) (Figure 3A). This suggests that PLSCR3 deficiency activates DNA sensing or cyclic dinucleotide synthesis in an mtDNA‐dependent manner. Under STING‐competent conditions, KD_PLSCR3 cells exhibited 28‐fold elevation in IFN*β* production (142.5 ± 11.3 vs. 5.1 ± 0.9 pg/mL; *p* < 0.0001) and 15‐fold increase in CXCL10 secretion (302.4 ± 18.6 vs. 20.1 ± 2.3 pg/mL; *p* < 0.0001) compared to NC (Figure 3B,C) in HT29 cells. Meanwhile, knocking down PLSCR3 also activated IFN*β* and CXCL10 secretion in DLD1 cells (Figure S4). CRISPR‐mediated STING knockout (STING sgRNA) completely abrogated these effects, with cytokine levels returning to baseline (Figure 3B,C). Western blot analysis confirmed upregulation of innate immune effectors in STING‐competent PLSCR3 knockdown cells, showing increased IFIH1, ISG60, phosphorylated STING (Ser366), and ISG15 protein levels relative to GAPDH loading controls (Figure 3D). Transcriptome profiling further demonstrated significant induction of interferon‐stimulated genes (ISGs) in these cells, including ISG15, ISG56, and ISG54 (Figure 3E). These data indicate that PLSCR3 deficiency–induced mtDNA release activates the IFN‐I response in CRC cells in a cGAS‐STING‐dependent manner.

### 3.4. PLSCR3 Deficiency Enhances the Sensitivity to Immune Cell–Mediated Killing in CRC

**Figure 4:**
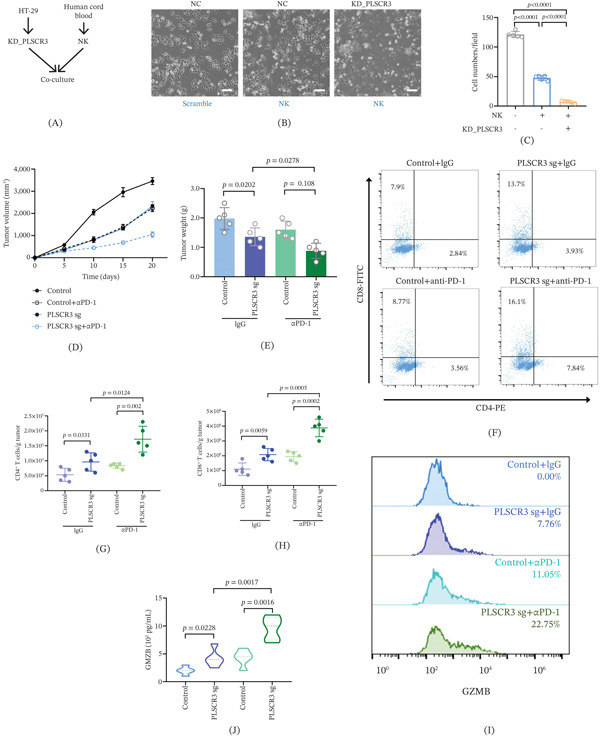
PLSCR3 deficiency enhances the sensitivity to immune cell–mediated killing in CRC. (A) Schematic of coculture system: PLSCR3 knockdown (KD_PLSCR3) HT29 cells or control were cocultured with human cord blood–derived NK cells at an E:T ratio of 5:1. (B, C) Phase‐contrast microscopy (scale bar: 50 *μ*m) showing morphological changes. Morphological analysis demonstrated decreased cell number in KD_PLSCR3 cells in the coculture condition (*p* < 0.0001, *n* = 5). (D) Mouse graft carcinoma with control or PLSCR3 knockout (PLSCR3 sg) CT26 cells were treated with or without anti‐PD‐1 antibodies (*α*PD‐1). Tumor growth curves (mm^3^) were recorded at Days 5, 10, 15, and 20 after tumor cell inoculation in immunocompetent BALB/c mice. (E) At Day 20, terminal tumor weights in the mouse graft carcinoma were measured. (F–H) Flow cytometry analysis of CD4+ and CD8+ tumor‐infiltrating T cells. The representative cell populations of CD4+ and CD8+ are shown. (I, J) Flow cytometry and ELISA analysis of Granzyme B (GZMB) expression (*n* = 5). Data are presented as mean ± SD. Tumor growth curves were analyzed using two‐way repeated‐measures ANOVA with post hoc multiple‐comparison tests. For multiple‐group comparisons, including panels I and J, one‐way ANOVA followed by Tukey′s post hoc test was used.

Multiple studies have demonstrated that activation of the endogenous cGAS‐STING pathway in tumor cells triggers both local and systemic immune responses, promoting the infiltration of various immune cell subsets into the TME and enhancing the cytotoxic function of NK cells [24, 25]. To further investigate whether targeting PLSCR3 can elicit effective antitumor immune activation via the cGAS‐STING pathway, we first examined the impact of PLSCR3 on NK cell cytotoxicity. PLSCR3‐deficient HT29 cells or control cells were cocultured with human cord blood–derived NK cells (Figure 4A).

Morphological analysis showed a reduced number of surviving PLSCR3‐knockdown cells under this coculture condition (Figure 4B,C). These data indicate that PLSCR3 knockdown increased the susceptibility of HT29 cells to NK cell–mediated killing under the tested E:T condition.

To identify whether PLSCR3 depletion induced an immune response in CT26 colon adenocarcinoma cells, mouse graft carcinoma with PLSCR3 knockout CT26 cells were treated using anti‐PD‐1 antibodies (10 mg/kg) at Days 6, 9, 12, 15, and 18 after tumor cell inoculation in immunocompetent BALB/c mice. Consistent with the previous report [26], mice injected with control CT26 cells were not sensitive to anti‐PD‐1 treatment. However, PLSCR3 silencing combined with anti‐PD‐1 treatment conferred a substantial inhibition of tumor growth in CT26 colon adenocarcinoma cells (Figure 4D,E).

Analysis of the graft TME by flow cytometry showed an increase in CD8+ and CD4+ T cell infiltration detected in the CT26 tumor graft after PLSCR3 depletion, which was strongly augmented by anti‐PD‐1 treatment (Figure 4F–H). Moreover, a minor to medium increase in GZMB expression and upregulation of activation of CD69, IFN*γ*, CD25, and CD107 were detected in tumor‐infiltrating CD8+ T cells from the CT26 tumor graft after silencing PLSCR3, which was strongly augmented by anti‐PD‐1 treatment (Figure S5).

Together, these data support that PLSCR3 depletion can increase tumor cell susceptibility to NK cell killing in HT29 cells and promote a more favorable tumor immune microenvironment during anti‐PD‐1 treatment in the subcutaneous CT26 model.

**Figure 5:**
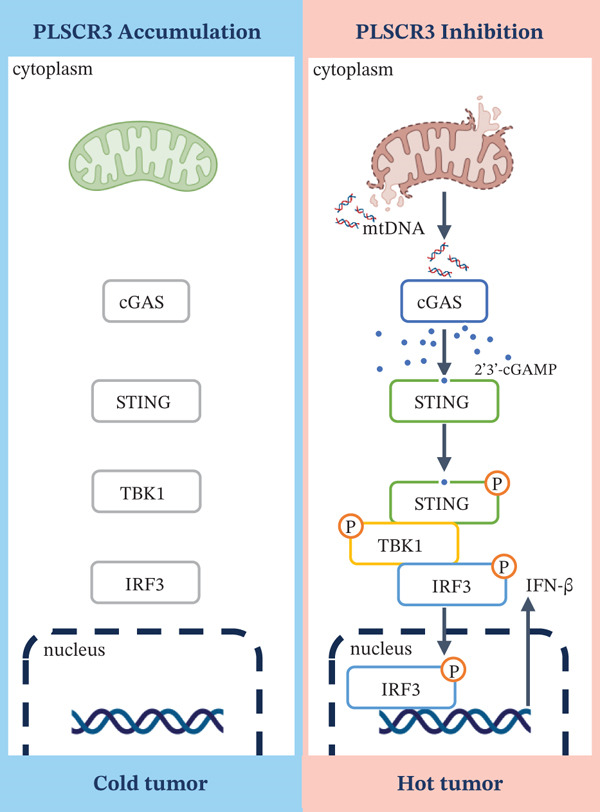
Schematic showing the mechanism by which PLSCR3 regulates mitochondrial integrity to modulate tumor immunogenicity.

Collectively, our data indicate that PLSCR3 sustains mitochondrial homeostasis to suppress cGAS‐STING signaling. As schematized in Figure 5, PLSCR3 ablation triggers mtDNA‐driven immunogenic conversion, providing a mechanistic basis for the enhanced anti‐PD‐1 efficacy observed in MSS‐CRC models.

## 4. Discussion

Our data support a role for PLSCR3 as a negative regulator of mtDNA‐associated innate immune signaling in CRC. PLSCR3 deficiency was accompanied by mitochondrial depolarization, impaired respiratory function, increased cytosolic mtDNA, and activation of the cGAS‐STING pathway. Functionally, these changes were associated with increased immune cell–mediated tumor killing and improved response to anti‐PD‐1 therapy in the CT26 model. These findings connect mitochondrial homeostasis to tumor immunogenicity in CRC.

Pharmacological agents frequently induce mitochondrial dysfunction to trigger mtDNA release and cGAS‐STING activation. Lovastatin promotes CRC cell apoptosis via mitochondrial oxidative stress–induced membrane depolarization and mtDNA release [27]. Oxaliplatin generates reactive oxygen species (ROS), opening mitochondrial permeability transition pores (mPTPs) to activate cGAS‐STING/TBK1/IRF5 signaling and immunogenic cell death [28]. In breast cancer, paclitaxel induces micronuclei formation and mtDNA leakage, activating cGAS‐STING to drive IFN and TNF*α* secretion that promotes apoptosis [29]. For endogenous regulators, OMM protein VDAC2 maintains membrane integrity during CD8^+^ T cell attack via IFN*γ*. VDAC2 inhibits BAK activation, preventing mitochondrial membrane collapse, whereas VDAC2 deficiency causes excessive BAK activation and mtDNA leakage [30].

PLSCR3 operates through a distinct mechanism. As an IMM phospholipid scramblase, it maintains membrane integrity independent of external signals. PLSCR3 deficiency autonomously triggers mtDNA leakage, revealing a fundamental innate immune regulatory process in CRC. Unlike VDAC2 deletion, which induces caspase‐dependent apoptosis, PLSCR3 deficiency primarily activates innate immunogenic signaling with minimal direct cytotoxicity (Figure 4). This establishes compartmentalized immunity across mitochondrial membranes: OMM proteins like VDAC2 regulate apoptosis‐driven immunity, while IMM proteins like PLSCR3 control innate immune responses through membrane stabilization.

mtDNA leakage following PLSCR3 deficiency may contribute to improved sensitivity to anti‐PD‐1 therapy in the CT26 subcutaneous MSS‐CRC model. Sustained low‐grade STING activation (Figure 3A,D) may bypass the epigenetic silencing and STING degradation that typically render some MSS tumors refractory to exogenous immune stimuli [31]. This intrinsic immunogenic reprogramming transforms immunologically “cold” MSS‐CRC into inflamed microenvironments, evidenced by expanded Granzyme B^+^ NK cells and enhanced CD8^+^ T cell infiltration (Figure 4F–J).

Recent studies have highlighted mtDNA release as a critical trigger of cGAS‐STING activation in cancer. Pharmacological agents such as oxaliplatin and ROS‐inducing compounds can promote mtDNA leakage and enhance antitumor immunity. In contrast to these exogenous inducers, our findings suggest that intrinsic mitochondrial regulators also play a key role in controlling mtDNA release. While OMM proteins such as VDAC2 have been implicated in apoptosis‐associated mtDNA release, our data indicate that IMM components such as PLSCR3 may regulate mtDNA retention under basal conditions. This positions PLSCR3 as a potential link between mitochondrial homeostasis and tumor‐intrinsic innate immune signaling.

PLSCR3 exhibits context‐dependent functionality. Although recognized as a pyroptosis amplifier in immune cells by facilitating cardiolipin exposure and GSDMD‐N‐terminal mitochondrial targeting [32], our data reveal an opposing role in CRC cells. PLSCR3 overexpression correlates with poor prognosis, while its ablation induces mitochondrial dysfunction, mtDNA leakage, and cGAS‐STING‐mediated antitumor immunity. This dichotomy stems from cell lineage–specific functions: Macrophages require PLSCR3 for acute mitochondrial permeabilization during immunostimulatory pyroptosis, whereas tumor cells exploit PLSCR3 to maintain mitochondrial homeostasis and evade chronic immunogenic stress. Mechanistically, PLSCR3 deficiency in CRC shifts cell fate from GSDMD‐mediated rapid lysis toward cumulative mtDNA‐driven immune activation—where persistent cGAS‐STING signaling supersedes attenuated pyroptotic responses. PLSCR3 thus functions as a pleiotropic regulator at the mitochondria–immune interface, with net effects on tumor progression determined by cellular context and mitochondrial damage kinetics.

Clinical translation demands precise therapeutic strategies. Although genetic PLSCR3 inhibition suggests therapeutic potential in the CT26 MSS‐CRC model (Figure 4D,E), potential on‐target toxicity in nonmalignant tissues necessitates tumor‐selective delivery approaches (e.g., antibody‐conjugated PLSCR3 siRNA). Low‐dose radiotherapy or chemotherapy combinations could locally disrupt mitochondrial membranes to synergize with anti‐PD‐1 while minimizing systemic toxicity. Key unresolved questions include (1) molecular mechanisms underlying PLSCR3‐mediated mtDNA stabilization in CRC, (2) modulation of PLSCR3 activity by common CRC mutations such as KRASG12D, and (3) whether pharmacological PLSCR3 inhibition recapitulates genetic ablation efficacy in additional CRC models, including orthotopic and patient‐derived xenograft/PDX‐derived organoid xenograft models. Addressing these questions will accelerate mitochondrial immunotherapy development.

In exploratory genomic analyses, PLSCR3 alterations appeared to be infrequent, suggesting that its contribution to CRC may be more closely related to expression level and mitochondrial function than to recurrent coding alteration. However, the relationship between PLSCR3‐dependent mitochondrial regulation and canonical CRC driver mutations or copy number states remains unclear and warrants further investigation.

This study has several limitations. First, the initial candidate prioritization and survival analyses were based on TCGA and KM plotter public datasets and were not validated in an independent GEO cohort or an institutional clinical cohort; therefore, the survival findings support association rather than independent prognostic value. Second, the in vivo validation used a single MSS‐CRC cell line, CT26, in a subcutaneous syngeneic graft model. More clinically relevant orthotopic models, patient‐derived xenograft/organoid xenograft models, and additional MSS‐CRC models will be required to determine the generalizability of the findings. Third, the NK cell killing assay was performed at a single E:T ratio (5:1) in human CRC cells and did not include a dose–response curve or CT26 validation; further studies across multiple E:T ratios and cell lines are needed to establish the breadth of immune‐regulatory effects. Fourth, although cytosolic mtDNA was quantified using differential fractionation, dedicated marker‐based assessment of fraction purity would further strengthen the interpretation. Finally, the relationship between PLSCR3‐associated mitochondrial regulation and common CRC driver alterations, such as APC, TP53, KRAS, or SMAD4 mutations, remains to be defined.

In summary, PLSCR3 functions as a mitochondrial quality control checkpoint that suppresses mtDNA‐driven cGAS‐STING activation and tumor immunogenicity in CRC. By connecting mitochondrial homeostasis to antitumor immunity and treatment response, this work identifies a novel targetable axis with significant therapeutic potential. Developing PLSCR3 inhibitors or modulators could yield effective combination strategies to enhance ICB for immunotherapy‐resistant CRC patients.

## Author Contributions

Limian Ling and Jingyu Wu contributed equally to this work and share first authorship.

## Funding

This study was funded by the National Natural Science Foundation of China, 10.13039/501100001809, 81701988, and the Key Research and Development Program of Zhejiang Province, 10.13039/100022963, 2025C02065.

## Ethics Statement

The study design was approved by the Second Affiliated Hospital of Zhejiang University School of Medicine (approval no: IRB‐2021‐562).

## Conflicts of Interest

The authors declare no conflicts of interest.

## Supporting information


**Supporting Information** Additional supporting information can be found online in the Supporting Information section. File S1: Figures S1–S5: This file contains the supporting information figures supporting the main results. Figure S1: PLSCR3 expression and localization in diverse tissues and cell lines. Figure S2: Genomic alteration landscape and clinical associations of PLSCR3. Figure S3: PLSCR3 deficiency disrupts mitochondrial integrity. Figure S4: PLSCR3 deficiency activates mtDNA‐dependent cGAS‐STING signaling. Figure S5: PLSCR3 deficiency enhances the sensitivity to immune cell–mediated killing in CRC.

## Data Availability

Publicly available datasets used in this study are available from TCGA, GTEx, and KM Plotter. The experimental data generated in this study are included in the article and its supplementary information or are available from the corresponding author upon reasonable request.
