# Supplementary material for: PLSCR3 Deficiency Triggers mtDNA‐Driven cGAS‐STING Activation to Potentiate Antitumor Immunity in Colorectal Cancer
Source: Hum Mutat. 2026 May 25;2026:8545428. doi: 10.1155/humu/8545428 (PMC13201902; doi:10.1155/humu/8545428)
Supplement: Supplementary file 1 — Supporting Information Additional supporting information can be found online in the Supporting Information section. File S1: Figures S1–S5: This file contains the supporting information figures supporting the main results. Figure S1: PLSCR3 expression and localization in diverse tissues and cell lines. Figure S2: Genomic alteration landscape and clinical associations of PLSCR3. Figure S3: PLSCR3 deficiency disrupts mitochondrial integrity. Figure S4: PLSCR3 deficiency activates mtDNA‐dependent cGAS‐STING signaling. Figure S5: PLSCR3 deficiency enhances the sensitivity to immune cell–mediated killing in CRC. [file HUMU-2026-8545428-s001.docx]

**
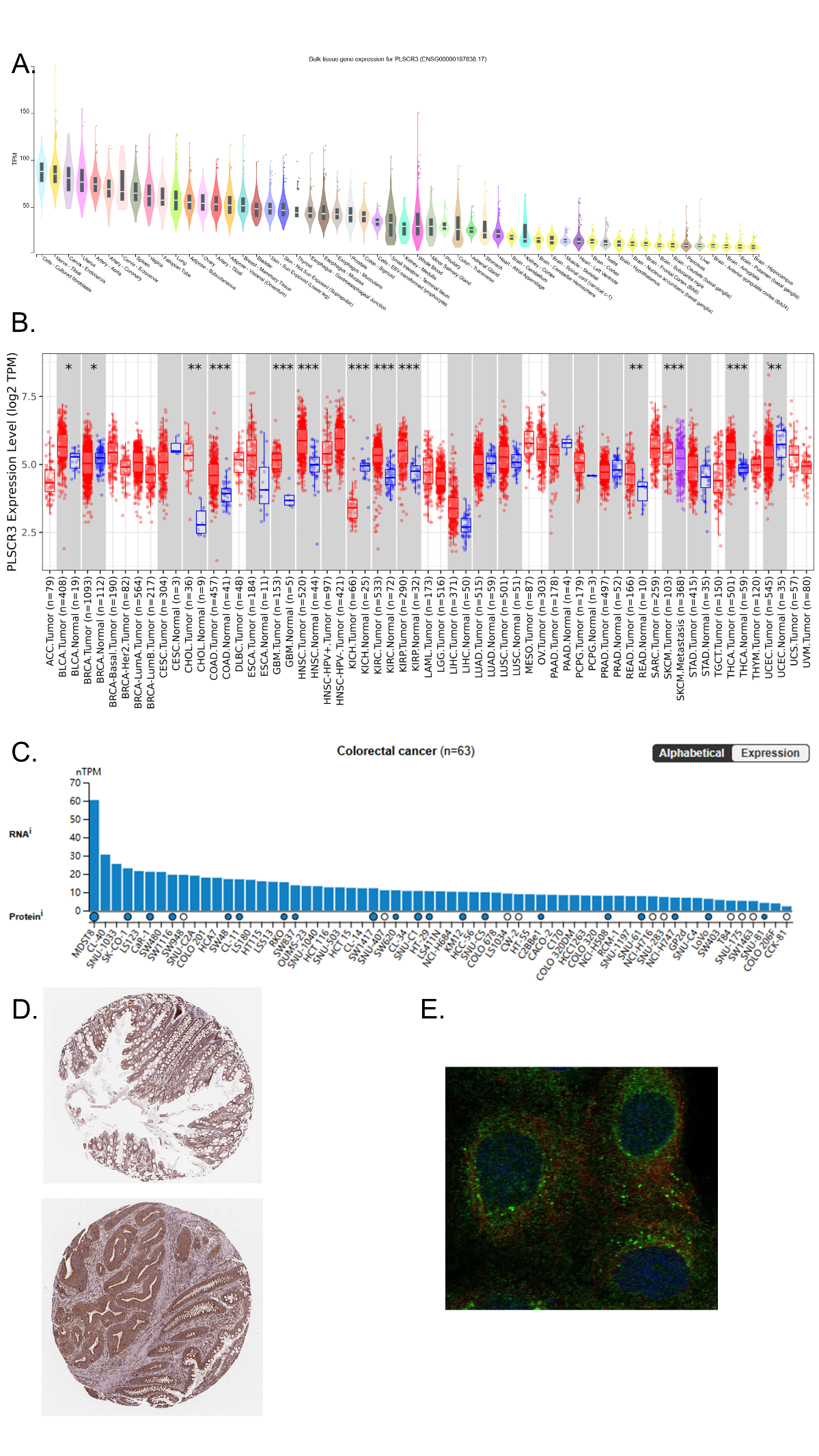
**

**Supplementary Figure 1.** **PLSCR3 expression and localization in diverse tissues and cell lines**.

(A) Violin-plot depicting PLSCR3 expression levels across various human normal tissues (https://www.gtexportal.org/). (B) Scatter-plot comparing PLSCR3 expression between normal tissues and matched tumor tissues (http://timer.comp-genomics.org/). (C) Box-charts presenting PLSCR3 expression at both RNA and protein levels in 63 colorectal cancer cell lines (https://www.proteinatlas.org/). (D) Immunohistochemical images illustrating PLSCR3 expression in normal colon (above, ID: 1857) and colon adenocarcinoma (below, ID: 4382) (https://www.proteinatlas.org/). (E) Fluorescence microscopy images showing PLSCR3 protein is localized in the mitochondria within human cell line A-431 (green fluorescence).

**
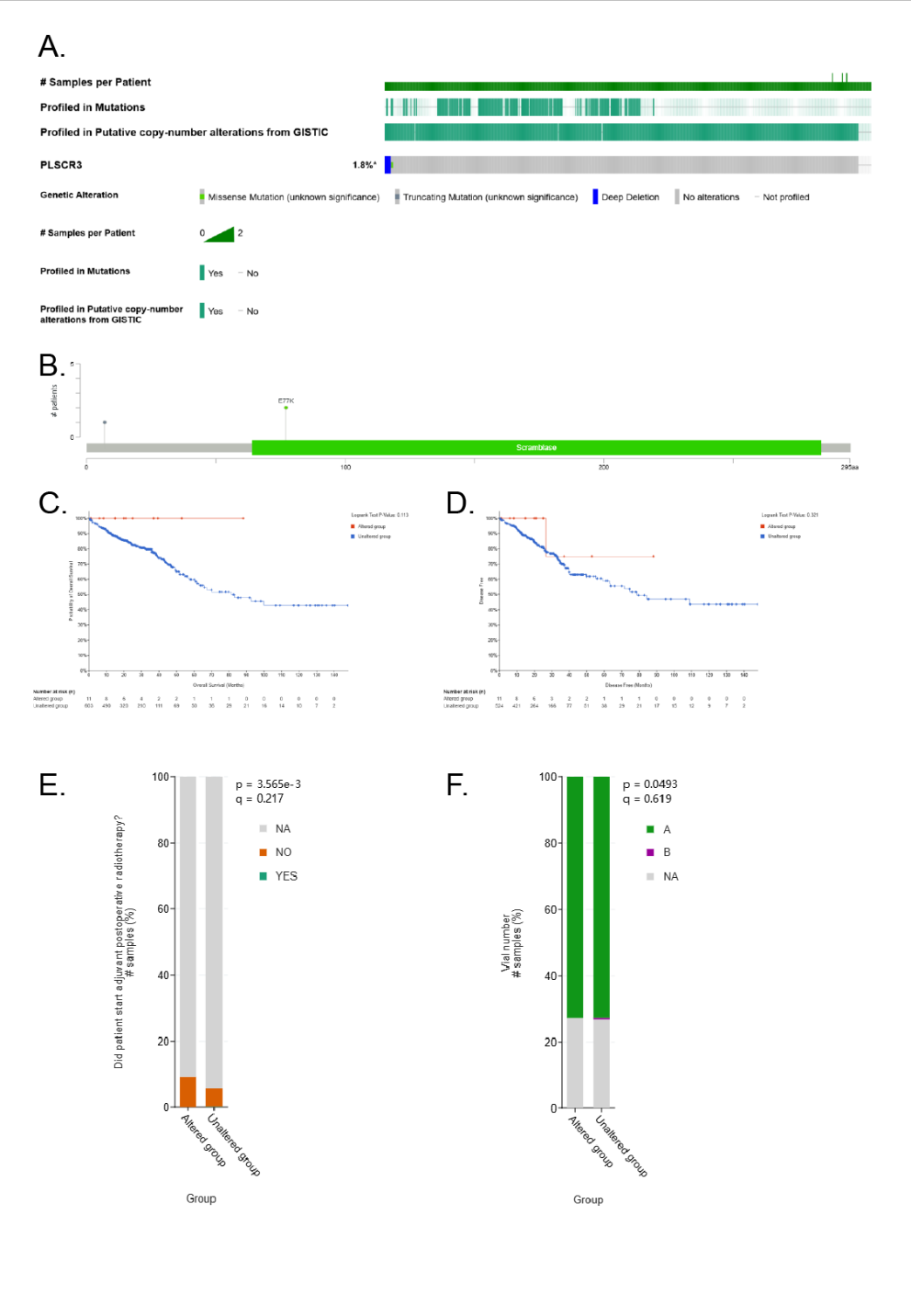
**

**Supplementary Figure 2. Genomic alteration landscape and clinical associations of PLSCR3.**

(A) Oncoprint showing the spectrum and frequency of PLSCR3 genomic alterations across the analyzed cohort, including missense mutations, truncating mutations, and deep eletions.

(B) Distribution of PLSCR3 mutations along the protein sequence. (C) Kaplan–Meier analysis of overall survival according to PLSCR3 alteration status. (D) Kaplan–Meier analysis of disease-free survival according to PLSCR3 alteration status. (E–F) Association analyses between PLSCR3 alteration status and selected clinical features in the analyzed dataset.

P values were calculated using the statistical methods implemented in the corresponding public analysis platform.

**
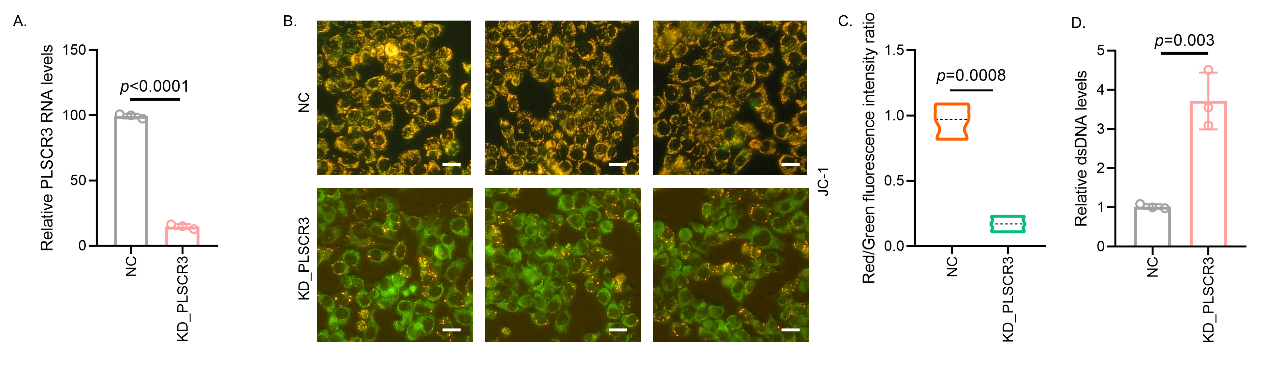
**

**Supplementary Figure 3.** **PLSCR3 deficiency disrupts mitochondrial integrity.**

(A) RT-PCR analysis verified reduction of PLSCR3 mRNA levels normalized to GAPDH compared to negative control (NC) in DLD1 cells (*p* < 0.0001, n = 3). (B) JC-1 staining (scale bar: 20 μm) showing green (depolarized mitochondria) and red (polarized mitochondria) fluorescence in DLD1 cells. (C) Quantification of the JC-1 red/green fluorescence intensity ratio. (D) Quantification of cytosolic double-stranded mitochondrial DNA (dsDNA) by qPCR in PLSCR3 knockdown cells and NC in DLD1 cells (*p*< 0.001, n = 3).


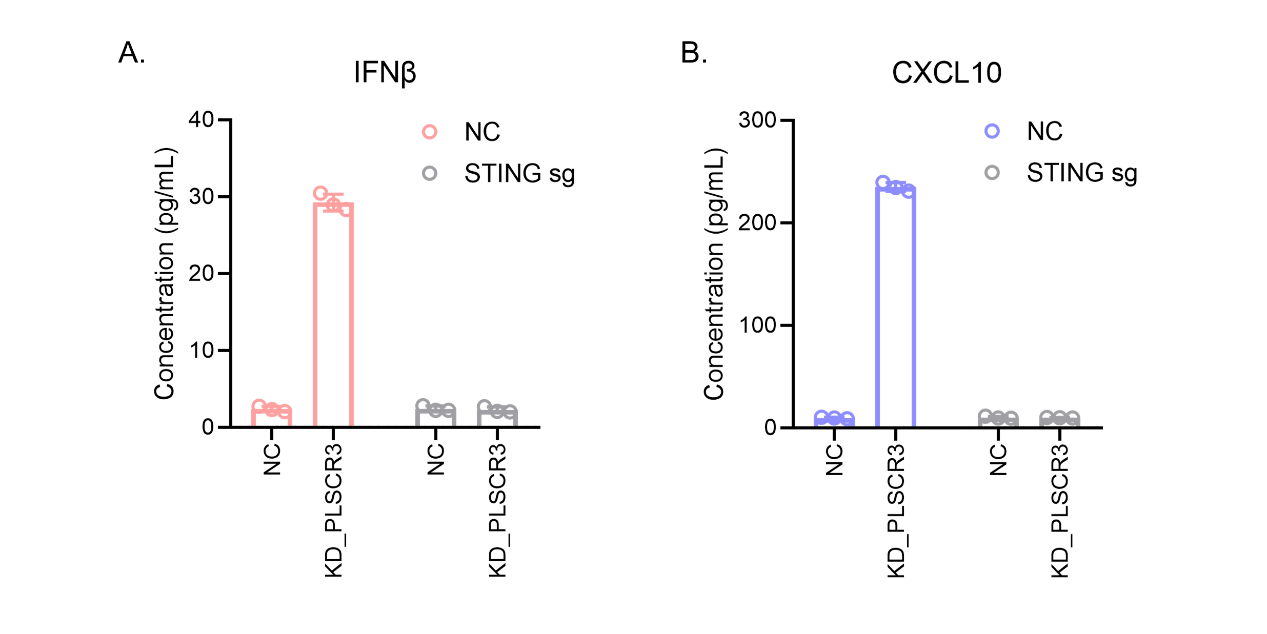
**Supplementary Figure 4. PLSCR3 deficiency activates mtDNA-dependent cGAS-STING signaling.**

(A-B) KD_PLSCR3 cells exhibited elevation in IFNβ and CXCL10 production measured by ELISA compared to NC in DLD1 cels (*p* < 0.0001, n = 3). CRISPR-mediated STING knockout (STING sgRNA) completely abrogated these effects.


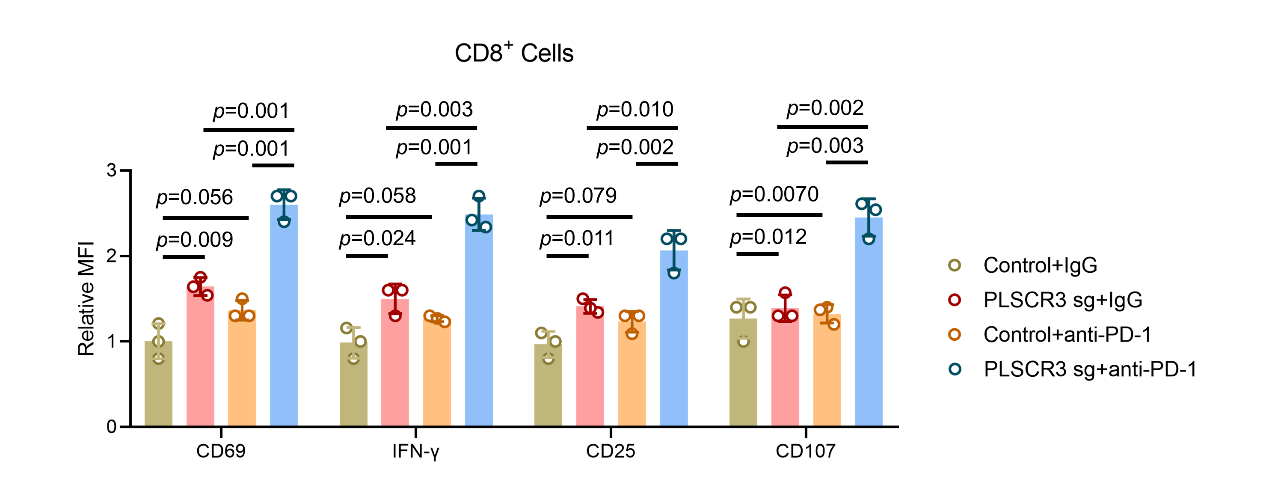
**Supplementary Figure 5. PLSCR3 deficiency enhances the sensitivity to immune-cell-mediated killing in CRC.**

Expression of activation markers of CD8+ T cells were measured by flow cytometry (n=3). MFI represents mean fluorescence intensity. Values represent mean ± SD. The unpaired, two tailed t-test.
